# Attitude and satisfaction of health care providers towards clinical pharmacy services in Ethiopia: A post-deployment survey

**DOI:** 10.1186/s40545-016-0058-6

**Published:** 2016-03-08

**Authors:** Arebu Issa Bilal, Zelalem Tilahun, Gebremedhin Beedemariam, Belete Ayalneh, Bisrat Hailemeskel, Ephrem Engidawork

**Affiliations:** Departement of Pharmaceutics and Social Pharmacy, School of Pharmacy, College of Health Sciences, Addis Ababa University, Addis Ababa, Ethiopia; Department of Pharmacology and Clinical Pharmacy, School of Pharmacy, College of Health Sciences, Addis Ababa University, P.O. Box: 1176, Addis Ababa, Ethiopia; Department of Pharmacy Practice, College of Pharmacy, Howard University, Washington, DC, USA

**Keywords:** Attitude, Clinical pharmacy services, Ethiopia, Health care providers, Perception, Post deployment survey, Satisfaction

## Abstract

**Background:**

Clinical pharmacy service has evolved steadily over the past few decades and is contributing to the ‘patient care journey’ at all stages. The service improves safety and effectiveness of medicines, thereby avoiding medication errors. As part of this global shift in pharmacy education and practice, Ethiopian Universities revamped the undergraduate pharmacy curriculum and the first graduates came out in July 2013. These graduates were immediately deployed in public hospital settings, with the ultimate aim of providing clinical pharmacy services. As such an initiative is new to the Ethiopian pharmacy sector, there is a need to do assessment of the health care providers’ perception and satisfaction towards the service.

**Methods:**

A cross-sectional survey using self-administered questionnaire was conducted in six regions and one-city administration of the country. Physicians, Health officers and Nurses working along with the new pharmacy graduates formed the study population. A total of 650 healthcare professionals participated in the study. Data were entered, cleaned and analyzed using appropriate statistical tools.

**Results:**

Majority of the health care providers agreed that clinical pharmacy service could have a significant contribution to the patient care. A large proportion of them (70–90 %) had a positive attitude, although there appeared to be some differences across professions. About 50 % of the professionals were of the opinion that patient care should be left to the health care providers and pharmacists should concentrate on drug products. In addition, the same proportion of respondents said that the setup in their respective hospital was appropriate for provision of clinical pharmacy service. Multivariable analysis indicated that attitude of the health care providers was significantly associated with year of experience.

**Conclusions:**

A large proportion of the health care providers had positive attitude towards the service, although the extent of the service was below their expectation. Hence, efforts should be in place to organize continuous professional training for pharmacists and awareness creation forums for other healthcare professionals.

## Background

Clinical pharmacy service is a patient oriented service developed to promote the rational use of medicines. More specifically, it is developed to maximize therapeutic effect, minimize risk, minimize cost and respect patients’ choice. The main activities performed by the clinical pharmacists include, among others, interacting with the health care team in patient rounds, interviewing patients and formulating medication histories, providing recommendations on drug selection, and follow-up of results to improve patient outcomes [1, 2]. Implementation of clinical pharmacy services and integration of pharmacists into core health care teams is instrumental in achieving better patient care, better team decision making and financial savings through cost-effective use of medicines and improved use of pharmaceutical expertise [3, 4].

There is variability in the degree of implementation of clinical pharmacy service among countries. Clinical pharmacy is well developed in some countries, but it is in early stages in others. In addition, there is disparity in the actual definition of clinical pharmacy throughout the world [5]. Nevertheless, the involvement of pharmacists in every step of the medication use process results in great returns, including increased patient safety, improvements in disease management and medicine therapy, improved effectiveness in healthcare spending, and improved patient adherence and quality of life [6]. In a study conducted at Jimma University specialized Hospital, 89.3 % of the interventions (total documented being 149) were initiated by clinical pharmacists, 49 % were rated to have major clinical importance and 68 % were fully accepted by physicians [7].

There is a body of evidence in the literature showing that physicians are receptive to several clinical services provided by pharmacists and had a positive overall attitudes if these services were provided in the form of consultations or in a supportive manner [8–10]. However, other studies reported the existence of physicians’ resistance to the role of clinical pharmacists, which might have been attributed to lack of physicians’ exposure to pharmacist participation in clinical activities [11–14]. Physicians’ acceptance of clinical pharmacists’ service depends on the value physicians attach to the service and their perception of the pharmacists’ competence [6, 7]. It is thus prudent to take note that proper implementation of clinical pharmacy service needs collaboration and communication between patients, pharmacists, physicians and other health care professionals.

Ethiopia has long been (47 years) known for its track record of product-oriented pharmacy education and practice [15]. Following revamping, harmonizing and implementation of the new clinical-oriented undergraduate pharmacy curriculum (in 2009/10 Academic Year); about 426 graduates came out of the public universities and deployed in public hospitals in August/September 2013. The deployment of these new graduates throughout the country marked the formal beginning of the clinical pharmacy service, even though few masters’ graduates and in-service trained pharmacists had already started the service in some hospitals. As a result, the graduates were expected to initiate clinical pharmacy service in settings where the service was non-existent and to expand the service where it had been initiated.

Although there are few institutional based studies regarding physicians’ expectation of clinical pharmacists, the status of clinical pharmacy service in the nation is unknown. The objective of this study was therefore to assess attitude and satisfaction of health care professionals in the implementation of clinical pharmacy service. In addition, the study also aimed at identifying factors that hinder such integration and suggest ways for their mitigation.

## Methods

### Study design

A cross-sectional survey was conducted in six regions and one city-administration of the country in September 2014. All hospitals in Ethiopia who employed the new pharmacy graduates as well as the health care providers (Physicians, Nurses and Health officers) who had been working in the selected health institutions formed the study population. Health officers are mid-level practitioners with a bachelor degree in public health. They provide promotive, preventive, curative and rehabilitative services including management and implementation of primary health care services as well as diagnosing and prescribing medicines to patients [16].

A total of 426 graduates came out in July 2013, and deployed in public hospitals across the country by the Ministry of Health according to the health service coverage and needs of the regions.

Hospitals that did not have pharmacy graduates of 2013, with the new undergraduate curriculum, were excluded from the survey. Health care providers that did not have the chance to work along with the new graduates were also excluded from the study. Of the 9 regions and 2 city administrations, six regions (Amhara, Harari, Oromia, Southern Nation, Nationalities and peoples region, Somali, and Tigray) and one city-administration (Addis Ababa) were selected purposively, taking into account the number of new graduates deployed. Accordingly, a total of 650 healthcare professionals were included from 32 General hospitals, 14 Referral hospitals and 5 specialized hospitals.

### Data collection procedure

The quantitative data was collected using a pre-tested self-administered questionnaire. The questionnaire was developed based on instruments used in previous studies and customized to the country’s context. The questionnaire had different sections that included information on health care providers’ sociodemographic characteristics as well as perception, satisfaction, contribution and limitations of current clinical pharmacy services.

A total of 12 postgraduate pharmacy students were recruited to assist in the data collection process. They attended a one-day training focused on the aim of the study and detailed review of the tools so as they could be able to provide explanations if needed. Besides the practical training, adequate supervision and follow up was done by the supervisors to maximize quality of the data collected.

### Data analysis

The collected data was then coded, entered, cleaned and analyzed using SPSS version 20. Health care providers attitude towards the role of the graduates was assessed on a five-point Likert scale ranging from 1 (Strongly Disagree) to 5 (Strongly Agree). In exploring the association of variables, the scale was then re-coded into Disagree (Strongly Disagree and Disagree) and Agree (Strongly Agree and Agree). Likewise, scores for satisfaction were also rated using a five point Likert scale of excellent, very good, good, satisfactory or poor. The scores were then recoded as satisfied (Excellent, Very good, Good and Satisfactory) and not-satisfied (Poor).

Simple descriptive statistics including mean, percentage and standard deviations were computed to summarize categorical variables. Moreover, multivariable logistic regression was performed to show possible associations between the dependent (items for attitude and satisfaction) and independent (age, sex, years of experience and marital status) variables and Statistical significance was set at *p* < 0.05.

### Ethical considerations

Ethical approval was sought from the Ethics Review Committee of the School of Pharmacy, Addis Ababa University (Ethical approval letter no ERB/SOP/01/07/2014). Permission was also granted from the selected health facilities and informed consent was obtained from each participant. Participants were also assured about confidentiality of the information obtained and were informed that the information used would be analyzed in aggregate.

## Results

A total of 650 questionnaires were distributed to the health care providers and 594 were collected, giving a response rate of 91 %. As depicted in Table 1, more than half (55 %) of the respondents were male and over 90 % were under the age of 35 years, with mean age of 28.2 (±6.6) years. Regarding total years of practice in the healthcare system, about two-third of the respondents had more than 2 years of experience, with Internal Medicine (41 %) and Pediatrics (30 %) being the two major practice sites for the respondents. Close to 60 % of the respondents were single and physicians (41.0 %) and Nurses with BSc (46.0 %) accounted for nearly 90 % of the respondents (Table 1).

**Table 1:** Demographic characteristics of the respondents (*N* = 594), September, 2014

Characteristics	Category	*N* (%)
Gender	Male	328 (55.0)
	Female	264 (45.0)
Age (in years)	<35	507 (90.0)
	≥35	55 (10.0)
Marital status	Married	230 (39.0)
	Single	353 (59.0)
	Divorced	5 (1.0)
	Widowed	5 (1.0)
Profession	Physicians	244 (41.0)
	BSc Nurses	275 (46.0)
	Health Officers	75 (13.0)
Current area of practice	Internal medicine	245 (41.0)
	Pediatrics	113 (19.0)
	Outpatient Department	76 (13.0)
	Surgery	40 (7.0)
	Gynecology/Obstetrics	36 (6.0)
	Emergency Medicine	31 (5.0)
	Psychiatry	17 (3.0)
	Others^a^	35 (6.0)
Total years of work experience in the healthcare system	<2 years	250 (43.0)
	2- 5 years	158 (27.0)
	>5 years	171 (29.0)
Total years of experience in setting where there is provision of clinical pharmacy service	<1 year	282 (82.0)
	≥1 year	62 (18.0)

^a^Includes: Oncology, Hematology, TB clinic

### Attitude of the health care providers’ towards clinical pharmacy services

Majority of the respondents (70–90 %) had a positive attitude towards clinical pharmacy services, although there appeared to be some differences across profession (Table 2). More than 80 % of the respondents agreed on the items clinical pharmacy service initiation is desirable in the Ethiopian health care system, clinical pharmacist participation in medical ward round is desirable, clinical pharmacist can improve over all patient outcomes, clinical pharmacist can monitor patient response to drug therapy, and also can play important role in patient education and counseling. In all items of attitude measurement, physicians tend to agree positively more than the health officers and nurses (Table 2). About 50 % of the professionals were of the opinion that patient care should be left to other health care providers and pharmacists should concentrate on drug products. In addition, the same proportion of respondents said that the setup in their respective hospital was appropriate for provision of clinical pharmacy service (Table 2).

**Table 2:** Attitude of health care providers towards clinical pharmacy services (*N* =594), September 2014

Attitude items	Respondents	Agree	Neutral	Disagree	AOR (95 % CI)
		*N* (%)	*N* (%)	*N* (%)	
Clinical pharmacist participation in medical ward round is desirable	Physicians	228 (43.02)	12 (41.38)	4 (11.43)	1.90 [0.17–20.39]
	Nurses	232 (43.78)	13 (44.83)	30 (85.71)	0.10 [0.01–0.88]*
	HO	70 (13.21)	4 (13.79)	1 (2.86)	1.00 [references]
	Total	530 (89.23)	29 (4.89)	35 (5.89)	
Clinical pharmacist can play important role in patient education and counseling	Physicians	230 (43.73)	10 (27.03)	4 (12.90)	0.92 [0.09–9.22]
	Nurses	224 (42.59)	25 (67.57)	26 (83.87)	0.15 [0.02–1.22]
	HO	72 (13.69)	2 (5.41)	1 (3.23)	1.00
	Total	526 (88.56)	37 (6.23)	31 (5.22)	
Clinical pharmacist can monitor patient response to drug therapy from toxicity/side effects perspective	Physicians	223 (42.97)	17 (37.78)	4 (13.34)	0.92 [0.09–9.22]
	Nurses	227 (43.74)	23 (51.11)	25 (83.33)	0.15 [0.02–1.22]
	HO	69 (13.29)	5 (11.11)	1 (3.33)	1.00
	Total	519 (87.37)	45 (7.58)	30 (5.05)	
Clinical pharmacist can monitor patient response to drug therapy from effectiveness perspective	Physicians	209 (41.97)	26 (43.33)	9 (25)	0.92 [0.09–9.22]
	Nurses	221 (44.38)	29 (48.34)	25 (69.44)	0.15 [0.02–1.22]
	HO	68 (13.65)	5 (8.33)	2 (5.56)	1.00
	Total	498 (87.88)	60 (6.92)	36 (5.06)	
Clinical pharmacist can provide drug information to health care professionals such as compatibility, stability, storage, availability	Physicians	231 (44.25)	10 (24.39)	3 (10.00)	1.82 [0.17–19.55]
	Nurses	222 (42.53)	27 (65.85)	26 (86.67)	0.17 [0.02–1.42]
	HO	70 (13.41)	4 (9.76)	1 (3.33)	1.00
	Total	522 (77.27)	41 (15.49)	30 (7.24)	
Clinical pharmacy service enhance patients appreciation and satisfaction	Physicians	209 (45.53)	30 (32.61)	5 (11.63)	0.41 [0.09–1.90]
	Nurses	184 (40.09)	56 (60.87)	35 (81.40)	2.89 [0.82–10.10]
	HO	66 (14.38)	6 (6.52)	3 (6.97)	1.00
	Total	459 (69.02)	92 (16.50)	43 (14.48)	
Clinical pharmacist should take patients medication history at admission	Physicians	186 (45.37)	38 (38.78)	20 (23.26)	1.33 [0.49–3.61]
	Nurses	159 (38.78)	57 (58.16)	59 (68.60)	0.29 [0.12–0.72]*
	HO	65 (15.85)	3 (3.06)	7 (8.14)	1.00
	Total	410 (74.20)	98 (15.68	86 (10.12)	
Clinical pharmacist should have access to patients chart and have a place to document their service	Physicians	194 (44.09)	37 (39.78)	13 (21.67)	0.61 [0.12–3.00]
	Nurses	185 (42.05)	45 (48.39)	45 (75.0)	0.14 [0.03–0.65]*
	HO	61 (13.86)	11 (11.83)	2 (3.33)	1.00
	Total	440 (74.20)	94 (15.68)	60 (10.12)	
Clinical pharmacist should analyses patient treatment and suggest changes of therapy when necessary	Physicians	193 (42.05)	32 (42.31)	18 (32.14)	0.38 [0.08–1.82]*
	Nurses	203 (44.23)	36 (46.15)	36 (64.29)	0.209 [0.04–0.93]*
	HO	63 (13.73)	9 (11.54)	2 (3.57)	1.00
	Total	459 (77.14)	77 (13.11)	58 (9.75)	
Clinical pharmacist should care about drug products and leave patient care to Doctors, Health Officers and nurses	Physicians	90 (30.51)	47 (45.19)	107 (55.16)	0.33 [0.16–0.68]*
	Nurses	160 (54.24)	45 (43.27)	70 (36.08)	0.77 [0.38–1.57]
	HO	45 (15.25)	12 (11.54)	17 (8.76)	1.00
	Total	295 (49.75)	104 (17.53)	195 (32.72)	
The current setup (infrastructure and environments of your hospital is appropriate for the provisions of clinical pharmacy service)	Physicians	107 (36.03)	64 (50.39)	73 (43.46)	0.60[0.29–1.23]
	Nurses	148 (49.83)	51 (40.16)	75 (44.64)	0.90 [0.45–1.80]
	HO	42 (14.14)	12 (9.45)	20 (11.99)	1.00
	Total	297 (50.17)	127 (21.45)	168 (28.38)	
Clinical pharmacy service initiation is desirable in Ethiopian health care system	Physicians	210 (44.03)	27 (35.06)	7 (17.95)	1.78 [0.40–7.85]
	Nurses	202 (42.35)	44 (57.14)	29 (74.36)	0.34 [0.09–1.30]
	HO	65 (13.63)	6 (7.80)	3 (7.69)	1.00
	Total	477 (80.44)	77 (12.98)	39 (6.58)	
Clinical pharmacist can improve over all patient outcome/quality of patient care	Physicians	215 (43.79)	28 (43.75)	1 (2.63)	17.80 [1.93–164.86]*
	Nurses	208 (42.36)	35 (54.69)	32 (84.21)	0.58 [0.19–1.70]
	HO	68 (13.85)	1 (1.56)	6 (13.18)	1.00
	Total	491 (82.79)	64 (10.79)	39 (6.41)	

Data was analyzed using multivariable regression; *p < 0.05

Multivariable analysis indicated that attitude of the health care providers was significantly associated with year of experience in three out of the nine attitude items (Table 3). Health care providers with 2 to 5 and >5 years of work experience had an odds of 6.707 (AOR = 6.707, 95 % CI [1.881–23.913]) and 2.761 (AOR = 2.761, 95 % CI [1.154–6.610]) agreement, respectively, for participation of the new graduates in medical ward rounds than those that had <2 years. Furthermore, those with an experience of 2 to 5 (AOR = 2.848; 95 % CI [1.021–7.947]) and >5 years (AOR = 3.419; 95 % CI [1.283–9.111]) had an almost equivalent rate (about 3-fold) of positive attitude for role of the pharmacists as drug information provider. However, it was only those with >5 years of experience that had a belief in the ability of clinical pharmacy services to enhance patient appreciation and satisfaction (AOR = 2.399; 95 % CI [1.020–5.641]) than those with <2 years of experience.

**Table 3:** Association of year of experience with attitude of health care providers on the roles of clinical pharmacists, September 2014

Attitude items	Years of work Experience	Agree; *N* (%)	Disagree; *N* (%)	AOR (95 % CI)
Clinical pharmacist participation in medical ward round is desirable***	<2 years	214 (41.3)	22 (64.6)	1.00
	2–5 years	147 (28.4)	3 (9.0)	6.70 [1.88–23.91]*
	>5 years	157 (30.3)	9 (26.4)	2.76 [1.15–6.61]*
Clinical pharmacist can provide drug information to health care professionals such as compatibility, stability, storage, availability***	<2 years	214 (42.0)	17 (56.7)	1.00
	2–5 years	138 (27.0)	6 (20.0)	2.84 [1.02–7.94]*
	>5 years	156 (31.0)	7 (23.3)	3.41 [1.28–9.11]*
Clinical pharmacist services enhances patients appreciation and satisfaction***	<2 years	194 (43.4)	22 (53.6)	1.00
	2–5 years	120 (27.0)	9 (22.0)	2.067 [0.86–4.97]
	>5 years	133 (30.0)	10 (24.3)	2.399 [1.02–5.64]*

*Data was analyzed by multivariable logistic regression; * *p* < 0.05; *** Indicates the presence of non–response by respondents; The following 6 items were found to be non–significant; *Clinical pharmacist can provide dose recommendations based on patient and drug factors; Clinical pharmacist should take patients medication history at admissions; Clinical pharmacist should have access to patients chart and have a place to document their services; Clinical pharmacist analyses patient treatment and suggest changes of therapy when necessary; Clinical pharmacist should care about drug products and leave patient care to doctors, HOs and nurses; Clinical pharmacy services initiation is desirable in Ethiopian health care system*

### Satisfaction of the health care providers

In assessing the difference in satisfaction regarding the service provided by the new graduates, over 70 % of the health care providers expressed their satisfaction for eight out of the twenty (Table 4, the first eight items) satisfaction items. Low scores were obtained for activities, including counseling of patients during discharge (55 %), documenting services in patient care (58 %); providing information on alternative drug regimen (58 %); assisting nurses in managing drug infusion rates (57 %) and in drug preparation (reconstitution, reformulation, dilution) (56 %).

**Table 4:** Level of satisfaction of the health care providers on the roles of clinical pharmacists by work experience, September 2014

Current Clinical Pharmacists’ Activities in their setting	Years of work experience	Satisfied; *N* (%)	Not satisfied; *N* (%)	AOR (95 % CI)
Clinical pharmacists provide timely information on drug availability***	<2 years	171 (44.0)	63 (43.4)	1.00
	2–5 years	103 (26.5)	45 (31.0)	0.72 [0.42–1.25]
	>5 years	115 (29.5)	37 (25.5)	0.50 [0.26–0.98]*
	Total	389 (73.0)	145 (27.0)	
Clinical pharmacists provide information on appropriate route of administration	<2 years	166 (43.0)	68 (47.5)	1.00
	2–5 years	111 (28.0)	37 (26.0)	1.11 [0.63–1.94]
	>5 years	114 (29.0)	38 (26.5)	0.64 [0.33–1.24]
	Total	391 (73.0)	143 (27.0)	
Clinical pharmacists participate in preventing, detecting and resolving any drug interaction	<2 years	164 (43.0)	70 (46.6)	1.00
	2–5 years	106 (27.0)	42 (28.0)	0.83 [0.48–1.43]
	>5 years	114 (30.0)	38 (25.3)	0.57 [0.29–1.11]
	Total	384 (72.0)	150 (28.0)	
Clinical pharmacist involve in side effect prevention and management	<2 years	167 (42.0)	67 (48.5)	1.00
	2–5 years	109 (27.5)	39 (28.2)	0.92 [0.53–1.58]
	>5 years	120 (30.3)	32 (23.2)	0.86 [0.43–1.70]
	Total	396 (74.0)	138 (26.0)	
Clinical pharmacists counsel patients regarding the safe & appropriate use of medications	<2 years	172 (43.0)	62 (46.3)	1.00
	2–5 years	111 (28.0)	37 (27.6)	0.89 [0.51–1.55]
	>5 years	116 (29.0)	35 (26.1)	0.61 [0.31–1.20]
	Total	399 (75.0)	134 (25.0)	
Clinical pharmacists participate in dose calculation for patients	<2 years	155 (42.0)	79 (50.0)	1.00
	2–5 years	108 (29.0)	40 (25.3)	1.01 [0.59–1.73]
	>5 years	113 (30.5)	39 (24.6)	0.68 [0.35–1.31]
	Total	370 (70.0)	158 (30.0)	
Overall satisfaction towards Clinical pharmacy services in your setting	<2 years	164 (44.0)	68 (44.4)	1.00
	2–5 years	108 (29.0)	38 (24.8)	0.99 [0.58–1.72]
	>5 years	103 (27.5)	47 (30.7)	0.57 [0.30–1.10]
	Total	375 (71.0)	153 (29.0)	
Clinical pharmacists advise about cost effective medication alternatives***	<2 years	111 (52.0)	40 (54.7)	1.00
	2–5 years	55 (26.0)	17 (23.3)	0.73 [0.33–1.58]
	>5 years	48 (22.4)	16 (22.0)	0.24 [0.08–0.68]*
	Total	214 (74.6)	73 (25.4)	
Clinical pharmacists provide information on alternative drug regimen	<2 years	118 (52.5)	33 (52.4)	1.00
	2–5 years	57 (25.3)	16 (25.4)	0.631 [0.284–1.402]
	>5 years	50 (22.2)	14 (22.2)	0.268 [0.094–0.762]*
	Total	225 (58)	63 (42)	
Clinical pharmacists actively participate in bed side discussions to assist clinicians on therapeutic care plan and drug selection	<2 years	90 (50.0)	60 (57.0)	1.00
	2–5 years	52 (29.0)	20 (19.0)	1.115 [0.550–2.259]
	>5 years	39 (21.5)	25 (24.0)	0.359 [0.144–0.890]*
	Total	181 (63.3)	105 (46.7)	

Data was analyzed by multivariable logistic regression; * *p* < 0.05; *** Indicates the presence of non–response by respondents; The following other 10 items were found to be not only non–significant but also the satisfaction score was less than 70 %; *Clinical pharmacists regularly present in the ward; Clinical pharmacists involve in drug preparation (reconstitution, reformulation, dilution); Clinical pharmacist counsel patients during discharge, Clinical pharmacists document their services in patient care; Clinical pharmacist identify and report adverse drug reaction; Clinical pharmacists participate in dose adjustment for pediatrics and patients with impaired renal and or liver function; Clinical pharmacists advice nurses on compatibility of IV drug administration/admixture; Clinical pharmacists advice nurses on storage/handling of drugs; Clinical pharmacists assist nurses in managing drug infusion rates; Clinical pharmacists actively participate in ward rounds with the health care team*

In an effort to establish the association between satisfaction and year of experience, multivariable regression analysis revealed that there was a negative association in three of the twenty items. Items related to drug information such as drug availability, cost-effectiveness, and alternative drug regimens were negatively associated with increasing year of experience, as the likelihood of getting such information was decreased by 2 to 5 fold in those >5 years than <2 years of experience (Table 4, the last two items).

The present study also revealed that there was variation in satisfaction rate by practice site (Table 5). The overall satisfaction rate ranged from 47.1 % (for Gynecology and Obstetrics) to 80.8 % (for Emergency Department). For item related to ward presence of the new graduates, the minimum satisfaction score (41.6 %) was reported by the health care providers working at the Department of Gynecology and Obstetrics. The maximum satisfaction score (92.3 %) was reported by staff at the Emergency Department for the item on advice provided by the new graduates on appropriate route of administration. For items presented to the nursing staff, advice on storage, handling and preparation of drugs achieved the lowest score (45.5 %) by nurses in the psychiatry ward. By contrast, nurses in the Outpatient Department gave the highest score (83.3 %) for the same item. Even though Internal Medicine and Pediatrics were the major wards where the graduates provided more clinical pharmacy service, items related to patient counseling during discharge and documentation of care given to patients were those rated with low satisfaction score by professionals working in these wards.

**Table 5:** Association of satisfaction score of the health care providers to the area of practice

	Ward	Satisfied (*n*, %)	Dissatisfied (*n*, %)	AOR (95 % CI)
Current Clinical Pharmacists Activities in your setting	Internal medicine	160 (70.0)	70 (30.0)	1.00
	Pediatrics	67 (61.0)	43 (39.0)	0.70 [0.43–1.14]
	Surgery	20 (53.0)	18 (47.0)	0.45 [0.22–0.93]*
	Emergency	16 (61.5)	10 (38.5)	0.67 [0.28–1.57]
	Outpatient department	35 (55.5)	28 (44.5)	0.55 [0.30–1.00]
	Gynecology/Obstetrics	15 (42.0)	21 (58.0)	0.30 [0.14–0.64]*
	Psychiatry	10 (59.0)	7 (42.0)	0.57 [0.20–1.60]
	Others	16 (55.0)	13 (45.0)	0.51 [0.22–1.17]
	Total	339 (62.0)	210 (38.0)	
Clinical pharmacists actively participate in ward rounds with the health care team	Internal medicine	170 (74.0)	60 (26.0)	1.00
	Pediatrics	66 (60.0)	44 (40.0)	0.551 [0.33–0.90]*
	Surgery	21(55.0)	17 (45.0)	0.39 [0.18–0.80]*
	Emergency	14 (54.0)	12 (46.0)	0.38 [0.16–0.89]*
	Outpatient department	34 (54.0)	29 (46.0)	0.37 [0.20–0.67]*
	Gynecology/Obstetrics	19 (53.0)	17 (47.0)	0.37 [0.18–0.79]*
	Psychiatry	14 (82.0)	3 (18.0)	1.51 [0.41–5.51]
	Others	15 (52.0)	14 (48.0)	0.36 [0.15–0.88]*
	Total	353 (64.0)	196 (36.0)	
Clinical pharmacists provide timely information on drug availability	Internal medicine	171 (74.0)	59 (26.0)	1.00
	Pediatrics	81 (74.0)	29 (26.0)	0.931 [0.55–1.57]
	Surgery	28 (74.0)	10 (26.0)	0.84 [0.38–1.88]
	Emergency	21 (80.8)	5 (19.0)	1.38 [0.49–3.87]
	Outpatient department	50 (79.0)	13 (21.0)	1.12 [0.56–2.25]
	Gynecology/Obstetrics	22 (61.0)	14 (39.0)	0.50 [0.23–1.05]
	Psychiatry	13 (76.5)	4 (23.5)	1.04 [0.32–3.37]
	Others	16 (55.0)	13 (45.0)	0.36 [0.15–0.82]*
	Total	402 (73.0)	147 (27.0)	
Clinical pharmacists provide information on the appropriate route of administration	Internal medicine	176 (76.5)	54 (23.5)	1.00
	Pediatrics	78 (71.0)	32 (30.0)	0.70 [0.41–1.18]
	Surgery	26 (68.0)	12 (22.0)	0.53 [0.24–1.16]
	Emergency	24 (92.0)	2 (8.0)	3.52 [0.80–15.49]
	Outpatient department	50 (80.0)	13 (21.0)	1.10 [0.53–2.25]
	Gynecology/Obstetrics	18 (50.0)	18 (50)	0.26 [0.12–0.56]*
	Psychiatry	10 (59.0)	7 (41.0)	0.38 [0.137–1.09]
	Others	22 (76.0)	7 (24.0)	0.74 [0.29–1.89]
	Total	404 (73.5)	145 (26.0)	
Clinical pharmacists counsel patients during discharge***	Internal medicine	135 (59.0)	95 (41.0)	1.00
	Pediatrics	54 (49.0)	56 (51.0)	0.66 [0.41–1.06]
	Surgery	22 (58.0)	16 (42.0)	0.733 [0.35–1.52]
	Emergency	15 (58.0)	11 (42.0)	0.901 [0.39–2.08]
	Outpatient department	29 (46.0)	34 (54.0)	0.500 [0.27–0.91]*
	Gynecology/Obstetrics	16 (44.0)	20 (56.0)	0.463 [0.22–0.97]*
	Psychiatry	13 (76.5)	4 (23.5)	2.094 [0.65–6.72]
	Others	16 (55.0)	13 (45.0)	0.746 [0.32–1.70]
	Total	300 (55.0)	249 (45.0)	
Clinical pharmacists participate in dose calculation for patients	Internal medicine	161 (70.0)	69 (30.0)	1.00
	Pediatrics	77 (70.0)	33 (30.0)	1.01 [0.60–1.69]
	Surgery	30 (79.0)	8 (21.0)	1.28 [0.54–3.04]
	Emergency	16 (61.5)	10 (38.5)	0.59 [0.25–1.41]
	Outpatient department	48 (76.0)	15 (24.0)	1.22 [0.62–2.43]
	Gynecology/Obstetrics	17 (47.0)	19 (53.0)	0.32 [0.15–0.68]*
	Psychiatry	13 (76.5)	4 (23.5)	1.16 [0.35–3.79]
	Others	24 (83.0)	5 (18.0)	1.48 [0.52–4.16]
	Total	386 (70.0)	163 (30.0)	
Clinical pharmacist identify and report adverse drug reactions	Internal medicine	159 (69.0)	71 (31.0)	1.00
	Pediatrics	71 (64.5)	39 (35.5)	0.80 [0.48–1.32]
	Surgery	22 (58.0)	16 (42.0)	0.44 [0.20–0.94]*
	Emergency	19 (73.0)	7 (27.0)	1.09 [0.43–2.76]
	Outpatient department	43 (68.0)	20 (32.0)	0.87 [0.45–1.66]
	Gynecology/Obstetrics	21 (58.0)	15 (42.0)	0.53 [0.24–1.14]
	Psychiatry	12 (71.0)	5 (29.0)	0.89 [0.29–2.78]
	Others	21 (72.0)	8 (28.0)	0.83 [0.33–2.04]
	Total	368 (67.0)	181(33.0)	
Overall satisfaction towards Clinical pharmacy services in your setting***	Internal medicine	171 (75.0)	57 (25.0)	1.00
	Pediatrics	74 (68.0)	35 (32.0)	0.66 [0.39–1.11]
	Surgery	25 (68.0)	12 (32.0)	0.56 [0.25–1.24]
	Emergency	21 (81.0)	5 (19.0)	1.26 [0.44–3.57]
	Outpatient department	44 (70.0)	19 (30.0)	0.72 [0.37–1.40]
	Gynecology/Obstetrics	16 (47.0)	18 (53.0)	0.24 [0.11–0.54]*
	Psychiatry	12 (71.0)	5 (29.0)	0.66 [0.21–2.02]
	Others	22 (76.0)	7 (24.0)	0.78 [0.30–1.99]
	Total	385 (71.0)	158 (29.0)	
Clinical pharmacists provide information on alternative drug regimens	Internal medicine	100 (80.0)	25 (20.0)	1.00
	Pediatrics	41 (73.0)	15 (29.0)	0.641 [0.29–1.38]
	Surgery	21 (87.5)	3 (12.5)	1.18 [0.31–4.48]
	Emergency	5 (71.0)	2 (29.0)	0.50 [0.08–2.90]
	Outpatient department	31 (77.5)	9 (22.5)	0.63 [0.24–1.62]
	Gynecology/Obstetrics	18 (64.0)	10 (36.0)	0.32 [0.12–0.83]*
	Psychiatry	4 (67.0)	2 (33.0)	0.33 [0.05–2.10]
	Others	10 (91.0)	1 (9.0)	1.60 [0.19–14.62]
	Total	230 (77.0)	67 (23.0)	
Clinical pharmacists advise about cost effective medication alternatives	Internal medicine	99 (80.0)	25 (20.0)	1.00
	Pediatrics	39 (70.0)	17 (30.0)	0.53 [0.25–1.14]
	Surgery	17 (71.0)	7 (29.0)	0.36 [0.12–1.07]
	Emergency	5 (71.0)	2 (29.0)	0.53 [0.09–3.08]
	Outpatient department	30 (75.0)	10 (25.0)	0.46 [0.18–1.14]
	Gynecology/Obstetrics	18 (64.0)	10 (36.0)	0.32 [0.12–0.82]*
	Psychiatry	4 (67.0)	2 (33.0)	0.31 [0.48–2.05]
	Others	8 (73.0)	3 (27.0)	0.35 [0.07–1.60]
	Total	220 (74.0)	76 (26.0)	
Clinical pharmacists participate in dose adjustment for pediatrics and patients with impaired renal and/or liver function***	Internal medicine	95 (77.0	29 (23.0)	1.00
	Pediatrics	38 (68.0)	18 (32.0)	0.62 [0.30–1.30]
	Surgery	15 (62.5)	9 (37.5)	0.27 [0.09–0.79]*
	Emergency	5 (71.0)	2 (29.0)	0.70 [0.12–4.08]
	Outpatient department	28 (70.0)	12 (30.0)	0.43 [0.18–1.04]
	Gynecology/Obstetrics	15 (54.0)	13 (46.0)	0.23 [0.09–0.60]*
	Psychiatry	3 (50.0)	3 (50.0)	0.16 [0.02–1.05]
	Others	9 (82.0)	2 (18.0)	0.68 [0.12–3.73]
	Total	208 (70.0)	88 (30.0)	
Clinical pharmacists actively participate in bedside discussions to assist clinicians on therapeutic care plans and drug selection***	Internal medicine	88 (71.5)	35 (28.5)	1.00
	Pediatrics	28 (50.0)	28 (50.0)	0.39 [0.20–0.78]*
	Surgery	16 (67.0)	8 (33.0)	0.58 [0.21–1.58]
	Emergency	3 (43.0)	4 (57.0)	0.28 [0.05–1.40]
	Outpatient department	25 (62.5)	15 (37.5)	0.47 [0.21–1.06]
	Gynecology/Obstetrics	16 (42.0)	12 (58.0)	0.41 [0.16–0.99]*
	Psychiatry	3 (50.0)	3 (50.0)	0.26 [0.04–1.55]
	Others	7 (64.0)	4 (36.0)	0.38 [0.09–1.49]
	Total	186 (63.0)	109 (37.0)	
Clinical pharmacists participate in preventing, detecting and resolving any drug interactions	Internal medicine	173 (75.0)	57 (25.0)	1.00
	Pediatrics	77 (70.0)	33 (30.0)	0.74 [0.44–1.25]
	Surgery	26 (68.0)	12 (32.0)	0.56 [0.26–1.23]
	Emergency	20 (77.0)	6 (23.0)	0.97 [0.36–2.57]
	Outpatient Department	45 (71.0)	18 (29.0)	0.71 [0.36–1.36]
	Gynecology/Obstetrics	21 (58.0)	15 (42.0)	0.40 [0.19–0.87]*
	Psychiatry	13 (76.0)	4 (24.0)	0.89 [0.27–2.90]
	Others	20 (69.0)	9 (31.0)	0.53 [0.22–1.29]
	Total	395(72.0)	154(28.0)	
Clinical pharmacists advise nurses on storage/handling of drugs	Internal medicine	69 (62.0)	43 (38.0)	1.00
	Pediatrics	36 (64.0)	20 (36.0)	1.02 [0.51–2.03]
	Surgery	8 (57.0)	6 (43.0)	0.76 [0.24–2.39]
	Emergency	12 (68.0)	6 (33.0)	1.24 [0.43–3.60]
	Outpatient department	20 (83.0)	4 (17.0)	4.11 [1.14–14.76]*
	Gynecology/Obstetrics	10 (83.0)	2 (17.0)	2.07 [0.40–10.69]
	Psychiatry	5 (45.5)	6 (54.5)	0.55 [0.15–1.93]
	Others	12 (66.7)	6 (33.3)	1.15 [0.39–3.38]
	Total	172 (65.0)	93 (35.0)	

Data was analyzed by multivariable logistic regression; * *p* < 0.05; *** Indicates the presence of non–response by respondents; The following other 16 items were found to be not significant**.** *Clinical pharmacists are involved in drug preparation (reconstitution, reformulation, dilution); Clinical pharmacists assist nurses in managing drug infusion rates; Clinical pharmacists advise nurses on the compatibility of IV drug administration/admixture ; Clinical pharmacists document their services in patient care; Clinical pharmacists counsel patients regarding the safe and appropriate use of medications; Clinical pharmacists are involved in side effect prevention and management*

Multivariable analysis was also performed to look for association between satisfaction and practice site. Accordingly, practice site had a negative significant association with satisfaction score for active participation in wards, except for Psychiatric ward. The likelihood for satisfaction was significantly less by 2 to 5 fold for Gynecology and Obstetrics staff compared to that of Internal Medicine for 45 % of the items analyzed. The second and third ranking wards where significant negative association found were Surgery (for 3 items) and Psychiatry (for two items). By contrast, Emergency Medicine was the only practicing site where positive association was found with the satisfaction element advising nurses on storage and handling of drugs (Table 5).

### Major contributions and limitations of the new graduates

**Fig. 1:**
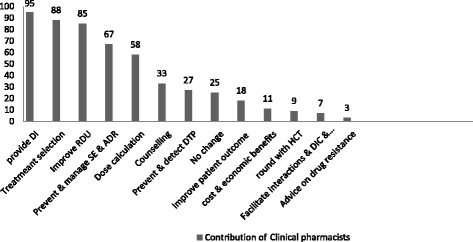
Major contributions of clinical pharmacists in the hospital health care activities, September 2014

The respondents were asked to list the major contributions of the graduates during their practice. Accordingly, provision of current drug information (16 %), assisting in selection of appropriate treatment (14.8 %), and improving rational drug use (14.3 %) were rated as the major contributions so far. The respondents also mentioned contributions like prevention and management of side effects and adverse drug reactions, and dose calculation as well as counseling (Fig. 1). Interestingly, 4.2 % of the respondents said that they did not see any change with implementation of the clinical pharmacy service.

**Fig. 2:**
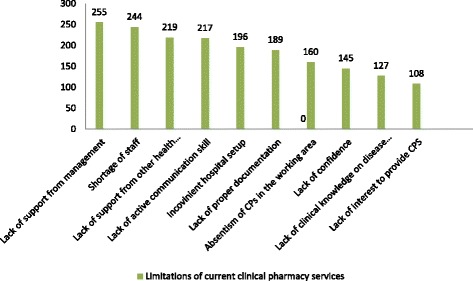
Limitations on current clinical pharmacy service from the health care providers perspective, September 2014

Among setbacks for rendering clinical pharmacy service, lack of management support (43 %), high work load (41 %) and lack of support from health care providers (36.8 %) ranked as the first three causes. Factors related to the pharmacists such as absenteeism (27 %), lack of effective communication skills (33 %) and lack of confidence (24.4 %) had also been cited as possible limitations (Fig. 2).

## Discussion

This study attempted to give a partial picture of the implementation of clinical pharmacy services in public hospitals found in Ethiopia. The study revealed that generally there is sufficient will amongst health care professionals to make the changes and accept the new roles assumed by pharmacists. The level of agreement in general appeared to be a function of the professionals’ year of experience, which is in line with a report that came out from West Virginia [8]. The present study also revealed that majority of the respondents had a positive attitude towards the changing role of pharmacists in their health care setting, which is concordant with a report from China [10], but discordant with that of Palestine [17]. According to the latter report, physicians in Palestine were more likely to accept traditional pharmacy services than newer clinical services.

In addition, differences were noted across profession. Nurses appeared to disagree more in some of the attitude items (for e.g., presence in the ward and taking medication history at admission) compared to Physicians and Health Officers. The reason behind this disagreement might be role conflict, which could possibly be sorted out with issuance of clear job description. Pharmacists should interact more positively and more frequently with the healthcare professionals in order to enhance their role [17]. Thus, Nurse’s disagreement on some issues might also be attributed to the existence of little or no interaction between the nursing and pharmacy staff.

Looking at assessment of satisfaction of the health care providers on performance of the new graduates, one could see that good performance was achieved in areas that are considered as traditional functions of pharmacists. In evolving roles such as counseling of patients during discharge, ward presence and active participation, and assisting nurses in drug preparation and administration; the rating was quite low. The low score in the new roles might be related to lack of inter-professional and communication skills as well as shortage of pharmacists assigned in the respective wards, as the impact of communication in improving interprofesstional collaboration is documented in other study [18]. Even if the score was low in this area, a greater proportion of the physicians had a positive attitude towards the extended role, in contrast to a study done in West Virginia, where physicians’ attitude towards pharmacists’ role in collaborative drug therapy management and pharmacists providing medication therapy management was not that favorable [8].

A growing body of evidence indicates that effective communication plays an essential role in provision of health care services [19]. Thus, the graduates and the educators should do more in the areas where deficiencies had been noted. Most of the new graduates were working in internal medicine and pediatrics wards as well as dispensary. This may be due to the high prevalence of drug related problems in internal medicine wards and vulnerability of the patient population in pediatrics ward. In fact, analysis of satisfaction taking practice site as a factor revealed that high satisfaction score was observed in most of the items in the two wards. This could show that assigning more pharmacists in these wards could have a significant impact on the clinical service. Indeed, the dissatisfaction observed in wards such as Surgery, Gynecology & Obstetrics, and Psychiatry, where little or no graduates were assigned, speaks for the importance of deploying more graduates. Interestingly, good satisfaction score was observed in most of the items in Emergency and outpatient Departments. The present study mirrors with other studies, which indicated that increasing presence of clinical pharmacists in such departments was crucial to increase patient outcomes [3, 8]. The graduates were also assigned in the dispensary due to shortage of pharmacists in this unit as well as because of the implementation of new systems (e.g., Auditable Pharmaceutical Transactions and Services, APTS). APTS is a service delivery arrangement enabling to establish a transparent and accountable medicines transaction and service provision system at health facilities that can be audited at any time [20]. Hence, the workload that was borne by the graduates would obviously decrease their performance in the ward since they spent more time in the dispensary, this is consistent with a study in Japan showing that a higher dispensing load was associated with fewer inpatients being provided clinical pharmacy services [21] and thus hospital management should look into possibilities of recruiting more graduates.

Documentation of pharmacy activities is an important component of clinical pharmacy services. The documentation of clinical pharmacy activities and the ability to identify pharmacists’ interventions are essential for assessing their impact on patient outcomes [22]. The fact that only 44 % of the surveyed hospitals documented the clinical pharmacy service they had provided strongly indicates that a system needs to be designed where services rendered could be documented and evaluated when the need arises. Lack of documentation and high work load have been reported elsewhere [4].

Though majority of the health care providers had a positive attitude towards clinical pharmacy service initiation in the Ethiopian health care system and believed that the service would improve overall patient outcomes, the professionals confessed that they did not extend the necessary support. Moreover, this problem is compounded by lack of support from the management side. The above-mentioned problems had been reported elsewhere in Nigeria [13], Pakistan [23] and Belgium [24]. On the other hand, it has been shown that collaboration among the various healthcare professionals with their respective expertise can lead to significant improvement in patient care [25]. Thus, hospitals should work more in the aspect of collaboration. It is also evident that without sufficient support from the management side, the program will not move forward to meet its intended goals. Hence, professionals and management should provide the necessary assistance so as the clinical pharmacy service could have a significant impact on patient outcomes.

The newly assumed roles involving direct patient care coupled to shortage of pharmacists is increasing pressure on pharmacist services, compromising patient outcomes [26]. In fact, many countries worldwide including Ethiopia experience shortage of pharmacists and the shortage is driven not only by limited resource but also by the changing role of pharmacists towards pharmaceutical care [27–30]. The shortage of the pharmacy workforce in Ethiopia is more severe than even other African countries, owing to the fact that Ethiopia has a low pharmacist density (2.38 per 100,000) compared to even the African average (8 per 100, 000) [31]^.^ This highlights the need for training more professionals that would be able providing better pharmaceutical care.

### Limitations of the study

This study might be subjected to recall bias as it is also true to all cross sectional surveys. In addition, the study was done shortly after clinical pharmacy service was implemented. Thus, health care providers might have limited time to observe and evaluate the clinical pharmacists’ performance which could possibly undermine performance of the graduate.

## Conclusions

A large proportion of the health care providers had a positive attitude towards the clinical pharmacy services, although the extent of the service was below their expectation. More than two-third of the health care providers had expressed their satisfaction in the services provided in their institution, although low scores for satisfaction were obtained in patient counseling during discharge and medication-related assistance extended to the nursing staff. Lack of support from management and health care providers, shortage of staff and lack of awareness on the role of pharmacists were identified as major challenges in the implementation of clinical pharmacy services in Ethiopia. Hence, a system should be in place to increase inter-professional relationships between the health care providers and pharmacists to enhance the role of pharmacists in improving patient care.
